# Role of Extracellular Loops and Membrane Lipids for Ligand Recognition in the Neuronal Adenosine Receptor Type 2A: An Enhanced Sampling Simulation Study

**DOI:** 10.3390/molecules23102616

**Published:** 2018-10-12

**Authors:** Ruyin Cao, Alejandro Giorgetti, Andreas Bauer, Bernd Neumaier, Giulia Rossetti, Paolo Carloni

**Affiliations:** 1 Institute of Neuroscience and Medicine (INM-9) and Institute for Advanced Simulation (IAS-5), Forschungszentrum Jülich, Wilhelm-Johnen-Strasse, 52425 Jülich, Germany; caobb0214@gmail.com (R.C.); alejandro.giorgetti@univr.it (A.G.); 2 Department of Biotechnology, University of Verona, Strada Le Grazie 15, 37134 Verona, Italy; 3 Institute for Neuroscience and Medicine (INM)-2, Forschungszentrum Jülich, 52428 Jülich, Germany; an.bauer@fz-juelich.de; 4 Institute for Neuroscience and Medicine (INM)-5, Forschungszentrum Jülich, 52428 Jülich, Germany; b.neumaier@fz-juelich.de; 5 Jülich Supercomputing Center (JSC), Forschungszentrum Jülich, 52428 Jülich, Germany; 6 Department of Oncology, Hematology and Stem Cell Transplantation, University Hospital Aachen, 52078 Aachen, Germany; 7 Department of Physics, RWTH Aachen University, 52078 Aachen, Germany; 8 Institute for Neuroscience and Medicine (INM)-11, Forschungszentrum Jülich, 52428 Jülich, Germany; 9 Department of Neurology, University Hospital Aachen, 52078 Aachen, Germany

**Keywords:** adenosine receptor, metadynamics, extracellular loops, allosterism

## Abstract

Human G-protein coupled receptors (GPCRs) are important targets for pharmaceutical intervention against neurological diseases. Here, we use molecular simulation to investigate the key step in ligand recognition governed by the extracellular domains in the neuronal adenosine receptor type 2A (hA_2A_R), a target for neuroprotective compounds. The ligand is the high-affinity antagonist (4-(2-(7-amino-2-(furan-2-yl)-[1,2,4]triazolo[1,5-a][1,3,5]triazin-5-ylamino)ethyl)phenol), embedded in a neuronal membrane mimic environment. Free energy calculations, based on well-tempered metadynamics, reproduce the experimentally measured binding affinity. The results are consistent with the available mutagenesis studies. The calculations identify a vestibular binding site, where lipids molecules can actively participate to stabilize ligand binding. Bioinformatic analyses suggest that such vestibular binding site and, in particular, the second extracellular loop, might drive the ligand toward the orthosteric binding pocket, possibly by allosteric modulation. Taken together, these findings point to a fundamental role of the interaction between extracellular loops and membrane lipids for ligands’ molecular recognition and ligand design in hA_2A_R.

## 1. Introduction

**Figure 1:**
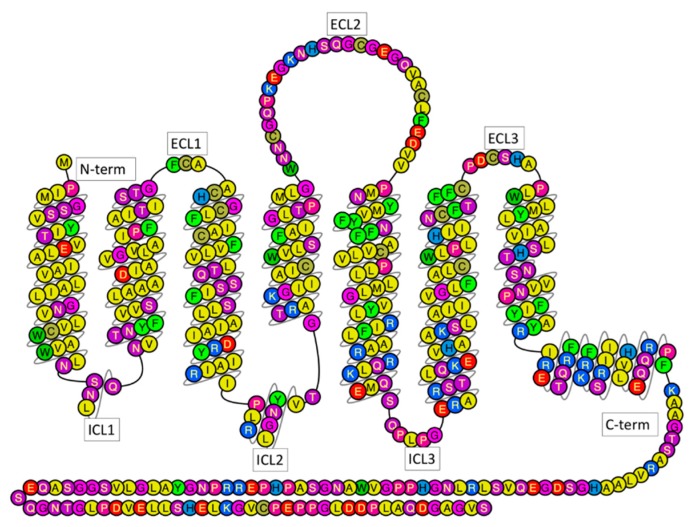
Snake view of hA_2A_R sequence, generated by GPCRDB [16]. Residues are colored differently depending on their polarity.

The human adenosine receptor type 2A (hA_2A_R, Figure 1) belongs to the human G protein-coupled receptors (GPCRs) [1], the largest membrane receptor family [2], essential for cell trafficking [3]. A_2A_R, highly localized in the striatum of the brain [4], is considered a promising drug target for combating Parkinson’s disease [5]. As in the other GPCRs, A_2A_R folds in seven transmembrane helices (H1 to H7), connected by three extracellular loops (ECL1 to ECL3) and three intracellular loops (ICL1 to ICL3). The N-terminus is extracellular, while the C-terminus is intracellular (Figure 1). Agonists and antagonists bind to the receptors’ orthosteric binding site (OBS), mostly from the extracellular space. The OBS is well extended into the hydrophobic core of the transmembrane bundles [6]. Agonist binding causes conformational changes of the receptor that ultimately lead to a variety of downstream processes.

A key role for ECLs in the early stages of molecular recognition of a variety of GPCRs is currently emerging [7]. They may influence ligand binding kinetics [8], serve as flexible gatekeepers along the ligand binding pathway [7,9], and act as selectivity filters against ligand subtypes [10]. ECLs may also contribute to the formation of an additional “vestibular” binding site (VBS) located well above the OBS [11,12,13,14,15].

**Figure 2:**
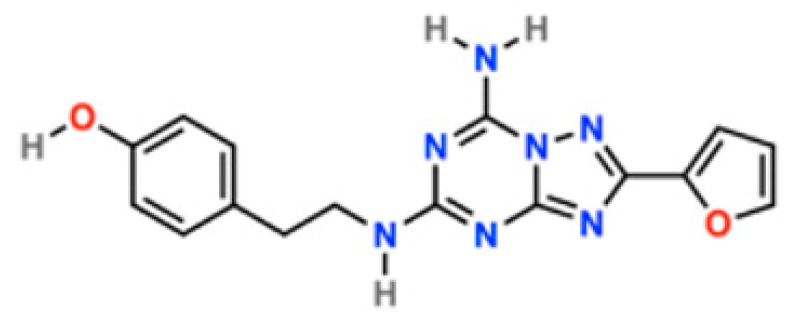
ZMA chemical structure, drawn with Maestro [33].

Hence, a detailed understanding of ECLs’ role for A_2A_R/ligand interactions may provide new opportunities for designing novel ligands targeting neurodegenerative diseases [5]. Here we explore that role through well-tempered metadynamics [17,18]. This is a simulation method that accelerates the sampling of specific degrees of freedom by adding a history-dependent potential term that acts on a small number of collective variables (CVs) [18,19]. Not only can metadynamics accurately predict the absolute ligand binding free energy [17], but it also reconstructs a multi-dimensional, CV-dependent free energy surface, from which receptor interaction sites and ligand binding poses, corresponding to local free energy minima, can be identified. We focus on the human adenosine receptor type 2A, in complex with its high-affinity antagonist ZMA ((4-(2-(7-amino-2-(furan-2-yl)-[1,2,4]triazolo[1,5-a][1,3,5]triazin-5-ylamino)ethyl)phenol) or ZM241385, Figure 2) [20]. The system appears to be suitable for this research for several reasons. First, the structural determinants of the complex are well known [21,22,23,24,25]. Next, a comparison with biophysical and computational studies [26] allowed us to establish the accuracy of our predictions. Finally, our computational setup—in particular the modeling of the membrane and the choice of the force field—was shown to be able to correctly reproduce ligand/receptor interactions [27]. In particular, the inclusion of a realistic membrane environment turned out to impact on the description of the molecular recognition events [27,28,29], here and in other GPCRs [30,31,32].

## 2. Results

**Figure 3:**
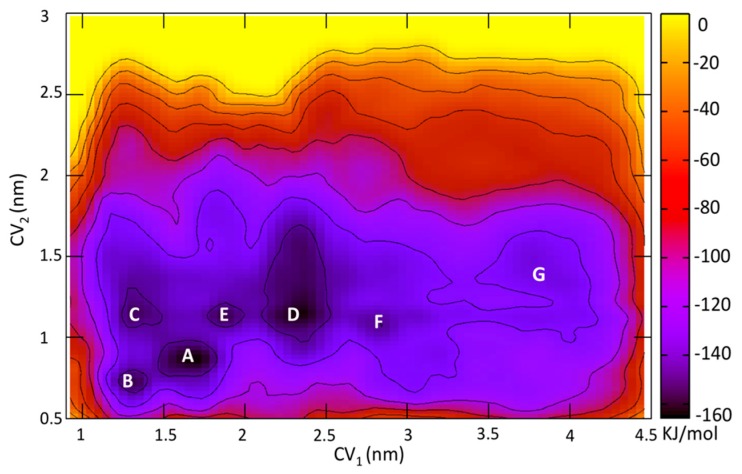
Free-energy surface associated with ZMA/hA_2A_R interactions, as a function of collective variables, CV_1_—a measure of ligand-OBS distance and CV_2_—a measure of the E169^ECL2^–H264^7.29^ distance. The figure shows the minima associated with the ligand located in the OBS **A**–**C**, in the vestibular binding site **D** in the salt bridge **E** and in a solvent-exposed moiety of the ECL2 **F**. In the OBS, the free energy in **B** and **C** are higher than that in **A** by 10.0 and 14.6 kJ/mol, respectively. **G** indicates the unbound state.

We performed well-tempered metadynamics simulations [17,18] to investigate the role of ECLs in ligand binding by reconstructing the free energy landscape of ZMA from the extracellular space to its fully bound form to the receptor (see Section 4 and Section S1 of the Supplementary Materials for further details). The free energy is calculated as a function of two apt collective variables (Figure 3). The first (CV_1_) has already been used to describe ligand binding/unbinding processes in GPCRs [19]. It is the distance between the centers of mass (COMs) of ZMA and the Cα atoms of the transmembrane helical bundles of hA_2A_R along the membrane’s normal axis. The second (CV_2_) takes into account the distance between H264^7.29^ and E169^ECL2^ at the entrance of the orthosteric binding site (OBS) of hA_2A_R. It is the distance between the Cα atom of E169^ECL2^ and the Cα atom of and H264^7.29^. These two residues can indeed form a salt bridge (see Section S2 and Table S1), which acts as a “gate” regulating the entrance of the ligand into the binding cavity [25,26]. The formation of this salt bridge is important for the ligand binding process [25,26]. Consistently, mutations of the residues in Ala and Gln impact the kinetics of unbinding [26]. During the 350-ns simulation, one cholesterol molecule binds to the hydrophobic cleft between helices H1 and H2, as previously observed [27].

The ligand bound to OBS in minimum **A** (Figure 3) represents the substate with the largest Boltzmann population, followed by minima **B** and **C**. However, the ligand turns out to also bind to an external or “vestibular” binding site (VBS), in a significant populated minimum (**D**). **D** is formed not only by helices’ residues but also by ECL1 and ECL2 residues along with lipid molecules. ECL2 might play an additional role in retrieving the ligand (**F** in Figure 3).

### 2.1. The Orthosteric Binding Site

**Figure 4:**
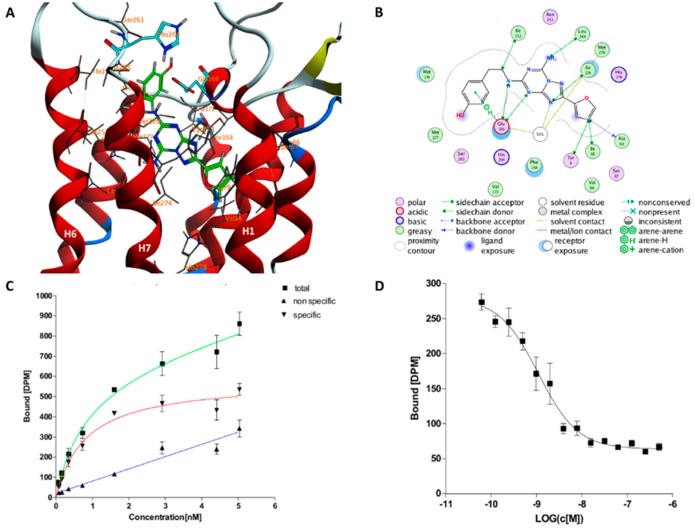
Lowest energy binding pose of ZMA in the orthosteric binding site (OBS, minimum **A** in Figure 3) in 3D (**A**) and 2D (**B**) representation. In (**A**) the protein backbone is render as cartoon, ZMA is shown as a green licorice, residues interacting with ZMA are shown as gray lines. The E169^ECL2^-H264^7.29^ salt bridge is shown in cyan licorice. Hydrogen, oxygen, and nitrogen atoms are specifically colored in white, red, and light blue, respectively. (**B**) 2D scheme of these binding pose in (**A**). Saturation binding assay result (**C**) and competition binding assay result (**D**) of ZMA/hA_2_AR complex as performed in this work. The other two binding poses of ZMA in **B** and **C** minima are shown in Figure S3.

The ensemble of conformations forming **A** correspond to the OBS in the X-ray structures (Figure 4). The free energy difference between **A** and the unbound state **G** is −79.5 kJ (see Section 4 and Appendix A for a definition of **G**). The standard state free energy (∆$G^{0}$) is calculated by taking into account: (i) The residual binding free energy on passing from the unbound state **G** to isolated ligand and receptor ($∆G^{Elec}$), this is estimated by solving the nonlinear Poisson‒Boltzmann equation [34] and (ii) The concentration of the protein in our simulation box (see Section 4). The calculated binding free energy without $∆G^{Elec}$ is 62.3 kJ/mol, with the correction is −58.2 ± 3.3 kJ/mol. This compares well with the experimental values found in the literature (K_d_ = 1.9 nM, ∆$G^{0}$ = −54.4 kJ/mol) [21] and that measured here (K_d_ = 0.8 nM, ∆$G^{0}$ = −54.0 kJ/mol, see lower panel of Figure 4). The ligand assumes an extended conformation similar to the ones present in other X-ray structures (Figure S1) with a Root Main Square Deviation (RMSD) lower than 0.24 nm of the Cα residues in the binding site (Figure S2). However, (i) the ligand’s bicyclic ring moiety flips by around 60 degrees relative to the initial binding pose; (ii) the ZMA’s furan ring moiety stretches towards H1 and H2, while it interacts with N253^6.55^ in this and other X-ray structures of the complex (see Figure S1). E169^ECL2^–H264^7.29^ salt bridge is present for 90% of the structures belonging to **A**. Consistently, this salt bridge is present in most PDB structures of hA_2A_R/ZMA complex (3EML [22], 4EIY [23], 3VG9 [24], 3VGA [24], 5UI7 [25], 5K2A/B/C/D [35], 5UVI [36], 5JTB [37], 5VRA [38], 6AQF [39] among others) except two (3PWH [21], 5NM2 [40]; see Section S2 and Table S1 for a complete list).

Minima **B** and **C** are higher in free energy by 10.0 kJ/mol and 14.6 kJ/mol. Here, the protein residues are less packed around the ligand: The volume of the OBS cavity increases from **A** to **B** and from **B** to **C** (0.38 nm^3^, 0.42 nm^3^, 0.45 nm^3^, for **A**, **B**, and **C**, respectively; see Table S2). The bicyclic core of ZMA in binding poses in **B** and **C** is more deeply extended into the OBS of hA_2A_R than in state **A** (see Figures S3 and S4). Interestingly, the E169^ECL2^–H264^7.29^ salt bridge interaction is formed in **B** but absent in **C** (99% and 2% of occurrence, respectively), as the Cα‒Cα distance between E169^ECL2^ and H264^7.29^ increases from 0.6 nm to 1.3 nm. This indicates that the E169^ECL2^–H264^7.29^ salt bridge interaction is affected by ligand binding in the OBS, as previously noted by Guo et al. [26].

### 2.2. Role of ECLs in Molecular Recognition

**Figure 5:**
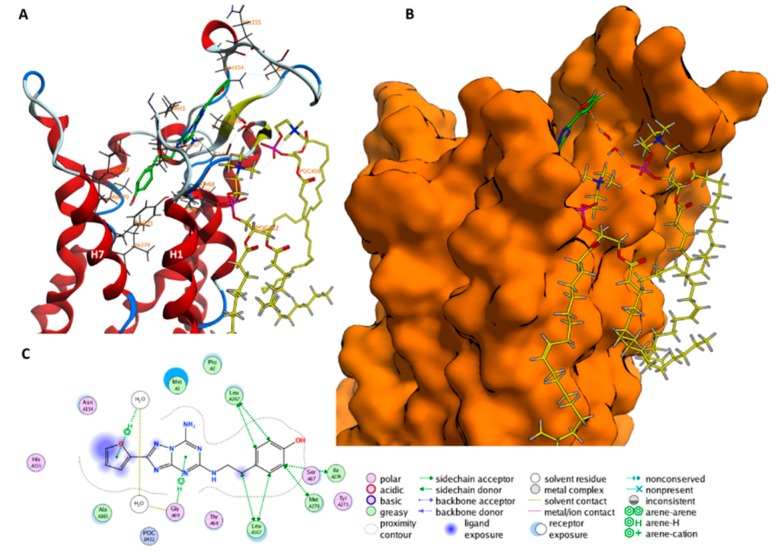
ZMA binding poses in the minimum **D** of Figure 3 is shown in the (**A**–**C**) panels as 3D, surface, and 2D representation, respectively. In (**A**) the protein backbone is rendered as a cartoon, ZMA and POPC molecules are shown as a green and yellow licorice, respectively, residues interacting with ZMA are shown as gray lines. Hydrogen, oxygen and nitrogen atoms are specifically colored in white, red and light blue, respectively. In (**B**) the solid protein surface, based on Van der Waal atom radii, is shown in orange.

Minimum **D** is higher in free energy than **A** by approximately two times k_B_T (≈4 kJ/mol). It is associated with two “vestibular” binding sites (VBS and VBS’ hereafter) located on the extracellular surface of the receptor at opposite sides of ECL2. Only the minimum associated with VBS is significantly populated (90% of the structures in **D**) and hence discussed here. Loops ECL1 and ECL2 form the VBS along with the extracellular ends of helices H1, H2, H7, and one lipid molecule (Figure 5). Lipids periodically find their way to that area and when the ligand is in the vestibular binding pocket, they establish water-mediated interactions. Two of the residues involved in ZMA binding, S67^2.65^ and L267^7.32^, in the VBS, if mutated, increase the residence time of ZMA for hA_2A_R by 1.5–2.3 folds, while showing negligible influence on the ligand binding affinity [26].

Among the residues that comprise the VBS, those located on ECL2, e.g., N154^ECL2^, H155^ECL2^ and A165^ECL2^, and H7, e.g., L267^7.32^, are not conserved across the human adenosine receptor subfamilies (Table S3). On the other hand, most of the residues located on the head of the remaining helices are better conserved, including Y9^1.35^ (100% conservation), E13^1.39^ (100% conservation), S67^2.65^ (75% conservation), M270^7.35^ (50% conservation), Y271^7.36^ (75% conservation) across the human adenosine receptor subtypes. Similar trend of conservation of these residues in A_2A_R across species is found (Table S3).

**Figure 6:**
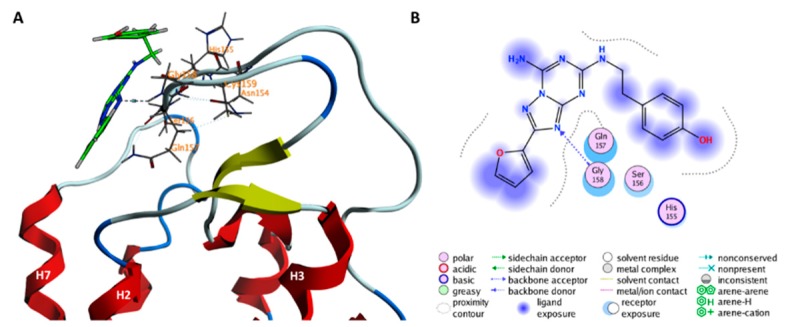
ZMA binding poses in the minimum **F** of Figure 1 are shown in the (**A**,**B**) panels, as 3D and 2D representation, respectively. In (**A**) the protein backbone is render as cartoon, ZMA is shown as a green licorice, residues interacting with ZMA are shown as gray lines. Hydrogen, oxygen, and nitrogen atoms are specifically colored in white, red, and light blue, respectively.

In the minimum **F**, ZMA interacts with a solvent-exposed motif of ECL2 (Figure 6): its 4-hydroxyphenyl moiety forms a hydrogen bond with E161^ECL2^, a water-mediated hydrogen bond with K150^ECL2^ and hydrophobic interactions with G152^ECL2^, K153^ECL2^, N154^ECL2^, H155^ECL2^ alkyl groups.

Although this minimum is not significantly populated (**F** is −20.92 kJ/mol higher in free energy than **A**), we suggest here it might play a role for ZMA’s binding to the receptor. Mutagenesis experiments found K153^ECL2^A mutation significantly decreased the dissociation rate of ZMA for hA_2A_R [26]. Mutations of two glutamic residues (E151^ECL2^ and E161^ECL2^) which are also located on the same solvent-exposed region of ECL2 have been shown to exert strong effects on ligand binding affinity [41]. The residues composing this solvent-exposed motif (K150–E161) are overall non-conserved (Figure S5) across the four human adenosine subfamilies. However, the conservation of the two glutamic residues is significant in A_2A_R across species (28% for E151^ECL2^ and 50% for E161^ECL2^, Figure S6).

### 2.3. An Access Control Binding Site

**Figure 7:**
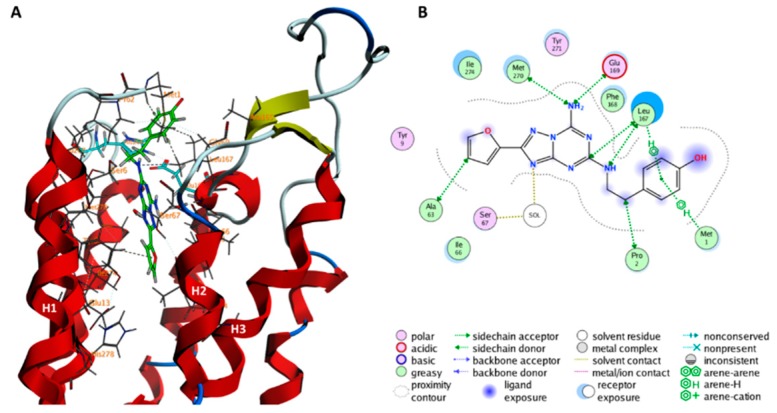
ZMA binding poses in the minimum **E** of Figure 3 is shown in (**A**,**B**) panels, as 3D and 2D representation, respectively. In (**A**) the protein backbone is render as cartoon, ZMA is shown as a green licorice, residues interacting with ZMA are shown as gray lines. The E169^ECL2^ and H264^7.29^ residues are shown in cyan licorice. Hydrogen, oxygen and nitrogen atoms are specifically colored in white, red and light blue, respectively.

In **E**, ZMA interferes with the E169^ECL2^–H264^7.29^ salt bridge (Figure 7) by H-bonding E169^ECL2^. The ligand additionally forms hydrophobic interactions with I66^2.64^ and water-mediated hydrogen bonding interaction with S67^2.65^, as in [26]. Consistently, H264^ECL2^A and E169^ECL2^Q variants [26] impact on a ligand’s dissociation rate, as do I66^2.64^A and S67^2.65^A variants on a ligand’s residence time in A_2A_R [26]. Interestingly, most of the residues involved in this binding site, specifically I66^2.64^, S67^2.65^, Y9^1.35^, M270^7.35^, and Y271^7.36^, correspond to a recently identified cryptic allosteric pocket [42]. The latter was suggested to be responsible for the selective binding of a novel bitopic antagonist against other adenosine receptor subtypes [42].

The E169^ECL2^–H264^7.29^ salt bridge is moderately conserved across human adenosine receptor subfamily members (see Section S3). Residues located in the lower region of the OBS are generally more conserved than residues located in the upper part of the binding pocket [43]. The first have been suggested to play a role for ligand affinity, the second for ligand specificity [43]. Here we demonstrate that the E169^ECL2^–H264^7.29^ salt bridge is better conserved in A_1_Rs and A_2A_Rs than A_2B_Rs and A_3_Rs (see Section S3). Granier et al. [10] has suggested that diversity in amino acid composition in the outer part the binding pocket may contribute to selection filter for larger ligands. Given these, we speculate that the gated access control site present at the binding pocket entrance of hA_2A_R, may be one important structural property that can selectively modulate ligands’ entering the OBS of adenosine receptor subtypes.

In conclusion of this section, let us analyze some common trends across the identified binding sites. As ZMA moves from the solvent-exposed minimum **F** to the membrane-facing vestibular minimum **D**, the number of water molecules decreases and the volume of OBS is the smallest (Table S2 number of water molecules around the ligand decreases from 33 (**F**) to 21 (**D**) while the volume of OBS increases from 0.33 nm^3^ to 0.34 nm^3^ (see Table S2). In minimum **E** and, even more in OBS, the number of water molecules decreases and OBS volume increases (Table S2). These trends are consistent with those uncovered in a microsecond-scale MD study [14] of the family A GPCR sphingosine-1-phosphate receptor [44].

### 2.4. Allosterism

We next asked ourselves whether the VBS and ECLs might act as allosteric sites for OBS. To address this issue, we focused on coevolving residues (residue pairs that are mutated in concert more frequently than random genetic events) between receptor binding sites and other protein regions [45,46,47]. Indeed, the latter are likely to play a role in the allosteric modulation of ligand binding [48].

**Figure 8:**
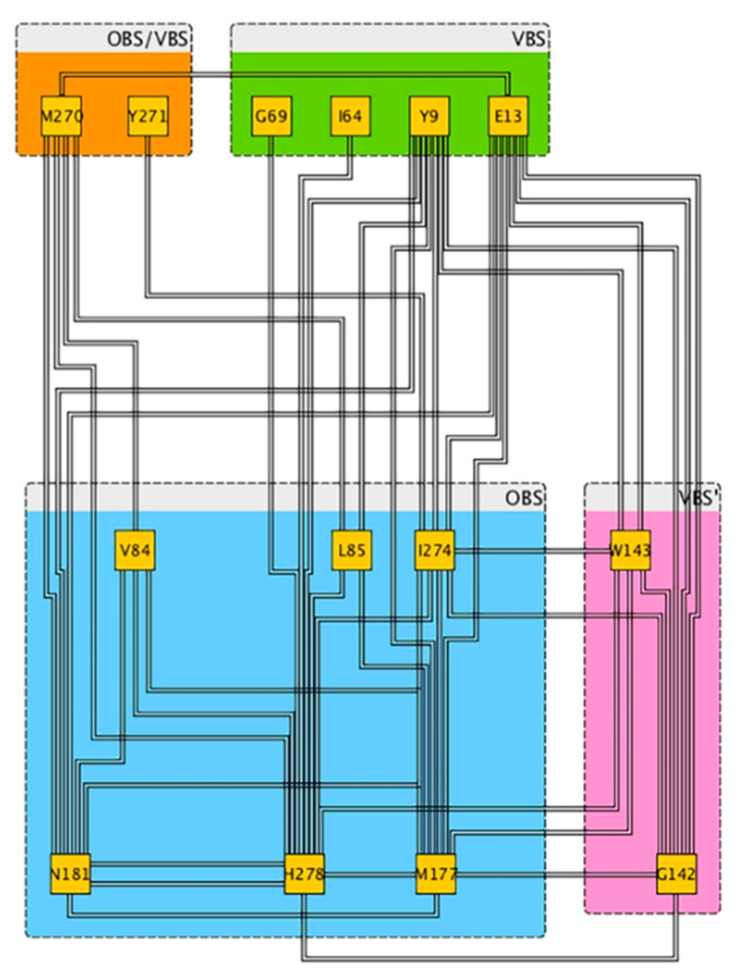
Coevolution relationships between amino acids of the relevant regions studied in this article (Table S4) based on Coeviz web server analyses [46].

The presence of allosterism is then identified by the so-called residue-paired coevolution score (PCS) [46]. The score ranges from 0 (no covariation) to 0.5 (moderate covariation) and 1 (complete covariation) [46]. In VBS, E13^1.39^ and Y9^1.35^ show moderate coevolutionary relation (PCS > 0.4) with residues located in the OBS, including V84^3.32^, L85^3.33^, N181^5.42^ and I274^7.39^ and H278^7.43^ (Figure 8 and Table S4). Also, G69^ECL1^ show moderate coevolutionary relation (PCS > 0.4) with H278^7.43^. Moreover, we find that M177^5.38^, I274^7.39^ and H278^7.43^ in the OBS [49] coevolve with G142^ECL2^ and W143^ECL2^ belonging to VBS’ (PCS 0.4–0.6). Interestingly, these two residues also coevolve with E13^1.39^ in VBS. Inspection of the structure allowed identifying a possible network of interactions connecting VBS and ECLs with OBS. E13 was shown to play a role in the stabilization of the UK432097 agonist and has been suggested to play a role in the on-rate ligand binding [50].

Hence, we conclude that ECL2 and VBS residues might be allosterically coupled with OBS. PCS analysis of the available crystallized 27 human GPCRs with OBS-bound ligand complex structures (subclasses A, B, C, and F) shows that, in the majority of them (22 structures), extracellular loops residues coevolve with OBS ones (Table S5). A section is offered in the Supplementary Materials on the sodium allosteric binding site (Section S4).

## 3. Discussion

Our metadynamics simulations have provided the free energy landscape of ZMA binding to hA_2A_R, embedded in a solvated neuronal-like membrane environment. Our calculations are consistent with the available experimental (i) and simulated (ii) data: (i) the predicted K_d_ is in agreement with that derived by measurements available in the literature [21] and performed in this work. (ii) The free-energy profile features a ‘multi-minima’ landscape, consistent with a multi-step dissociation process suggested by previous temperature accelerated molecular dynamics simulation [26]. In particular, the residues interacting with the ligand in [26] are the same ones in our minima.

The ligand is located in the OBS as in X-ray structure [21] (minimum **A** in Figure 4), but it slightly differs in the orientation of its bicyclic ring. This might be ascribed, at least in part, to the dramatic differences in the protein environment. Indeed, the environment changes from a detergent micelle of the X-ray structure [21], to a membrane-mimicking environment, rich in cholesterol, in the MD [27,51]. Notably, cholesterol binds to a pocket located between helices H1 and H2 (see Figure 5 in [27]). This may affect ligand binding poses (via an allosteric mechanism [27]) and affinity [30] in GPCRs.

Our metadynamics simulations further reveal the existence of significantly populated states (minima **B**, **C** and **E**), where ZMA interferes with the E169^ECL2^–H264^7.29^ salt bridge located between ECL2 and ECL3 of hA2AR. In minima **B** and **C,** the ligand is still in the OBS but in **B**, the phenol moiety of ZMA form a hydrogen bond with E169^ECL2^, possibly weakening the electrostatic strength of the salt bridge, while in **C** the phenol moiety is exposed toward the solvent and the salt bridge is broken. Notably, despite the fact that the geometrical position of ZMA is very similar (see Figure S4), the free energy increases on passing from **B** to **C** with respect to **A**, pointing toward a key role of such salt bridge in controlling the dissociation kinetics of ligands, as also suggested in [8]. Accordingly, H264^7.29^A and E169^ECL2^Q mutations impact on the ligand’s dissociation rate [26]. In **E**, ZMA is located between the OBS and the VBS and, although the E169^ECL2^–H264^7.29^ is formed, its electrostatic strength is possibly decreased by a hydrogen bond of the ligand’s triazin moiety with E169^ECL2^. In this binding site, ZMA also interacts directly with I66^2.64^ and forms a water-mediated hydrogen bonding interaction with S67^2.65^. These residues, if mutated in alanine, I66^2.64^A and S67^2.65^A, impact on the ligand’s residence time in A_2_AR [26].

The E169^ECL2^–H264^7.29^ salt bridge therefore seems to act as a “narrowing gate”, similar to what was observed in human GPCR β_2_ adrenergic receptor. Here, the D192^ECL2^–K305^7.32^ salt bridge acts as a gate and the salt bridge is located at the entrance of the OBS of this receptor, deeply buried in the transmembrane region [11,52].

In addition to these minima, the extracellular loops ECL1, ECL2, as well as the heads of helices H1, H2, H7, contribute to the formation of a previously unnoticed, significantly populated vestibular binding site (VBS) accommodating the ligand (minimum **D**). Consistently, mutating S67^2.65^ and L267^7.32^, two VBS residues, increase the residence time of ZMA for hA_2A_R by 1.5–2.3-fold, while showing negligible influence on the ligand binding affinity [26]. The role of some residues in ECL1 ECL2 of hA_2_A in ligand binding affinity was already shown elsewhere [53], and the discovery that ECL2 forms the VBS is in line with several studies on other GPCRs [11,12,13,15]. However, we suggest here that such VBS is not transient but is actually significantly populated. Interestingly, the recently resolved structure of hA_2A_R in complex with 5-amino-*N*-[(2-methoxyphenyl)methyl]-2-(3-methylphenyl)-2*H*-1,2,3-triazole-4-carboximidamide (Cmpd-1, hereafter) identified one potential allosteric pocket [42] that is located in the helical part of the VBS, as the one identified from our metadynamics simulation. Specifically, the site accommodating the methoxyphenyl group of Cmpd-1 consists of Y9^1.35^, A63^2.61^, I66^2.64^, S67^2.65^, L267^7.32^, M270^7.35^, Y271^7.36^ and I274^7.39^, five of which are located in the helical part of the VBS (see Figure 5).

A further interesting feature of our identified VBS, is that a lipid molecule contributes to the stabilization of the ligand binding. A key role of lipids for ligand binding has been already pointed out in other studies (see, for instance, [54,55]). The lipid bilayer was found to form the determinant entry pathway along which the ligand gains access to GPCRs [56] and even form a “membrane vestibule” that controls ligand binding kinetics [14]. Lipid composition in membrane could also modulate stability of specific ligand binding pose [27,28]. Therefore, altered lipid composition in the neuronal membrane could affect ligand binding. This, in turn, could alter the function of the receptor [57,58].

ECL2 forms a third binding site on the solvent-exposed region of the receptor, topographically distinct from the VBS (minimum F). The existence of the site might be consistent with mutagenesis experiments [26,41], since mutations of residues E151^ECL2^, K153^ECL2^, and E161^ECL2^, which are found to directly interact with ZMA in our simulations, significantly influence ligand binding affinity or dissociation speed. At the speculative level, we suggest that ECL2 might function as a ‘fishing’ moiety for the ligand in the extracellular compartment, redirecting it toward the VBS, consistent with the fact that the volume of the OBS increases upon ligand binding from ECL2 to the OBS.

Specific residues belonging to VBS or located in the ECLs, turn out to co-evolve with residues in the OBS, suggesting an allosteric pathway connecting the extracellular domains of the receptor to OBS (Figure 8). The pathway might impact on ligand binding. A similar conclusion was reached for another GPCR, the dopamine D_2_ receptor. For the latter, coevolved residues pairs show functional coupling in controlling responses to dopamine [59].

We close this section by analyzing major limitations of this work. First, experimental evidence indicates that hA_2A_R can form homo- and/or hetero-assemblies of two or more monomers [60,61]. It is expected that oligomeric order and architecture of the supramolecular assembly, not considered here, may affect ligand binding [62]. Second, the prediction of energetics and binding poses is determined by a priori choice of a set of CVs [19,63]. In this case, this issue might be alleviated by the fact that a wide range of optimal CVs are available to describe ligand binding to a GPCR [19]. Third, the calculated absolute ligand binding free energy might contain a significant source of inaccuracy from the use of necessarily approximate force fields [64] as well as nonlinear Poisson–Boltzmann calculations [34]. Fourth, the level of theory we employed inherently neglect the electronic degrees of freedom, that might be relevant for ligand binding. However, in this case no covalent binding occurs and therefore the polarization effects are negligible. Fifth, other components of cellular membrane, such as polyunsaturated chains and sphingolipids, have not been included in our membrane model [27]. The content of these is far less than cholesterol, however, also these biomolecules might impact on GPCRs function [65]. Finally, the sodium ion, recently discovered in the high-resolution structure of hA_2_AR [66], was not considered here. The consistency with experiments makes us suggest that these issues do not affect substantially the main predictions of the paper, namely the contribution of ECL2 to two significantly populated binding sites other than the OBS, along with the key role of lipids for the molecular recognition process.

## 4. Materials and Methods

### 4.1. System Preparation and MD Simulations

We have shown elsewhere that the conformation of the hA_2A_R is affected by membrane composition [27]. One of the main players in membrane-driven modulation of hA_2A_R is cholesterol, that specifically binds in a cleft between H1 and H2 and can allosterically affect the shape of the orthosteric binding site (OBS). Despite the fact that in cellular membranes, cholesterol content varies from 33% to 50% [67], unfortunately, cholesterol-driven allosteric effects are not captured in X-ray structures since artificial detergent-based environment or solubilizing antibodies are used [68]. In an effort to model hA_2A_R in a membrane environment mimicking the real cellular membrane, we embedded the receptor in a membrane of 42% POPC, 34% POPE and 25% of cholesterol molecules, mimicking the ratio among the three components in human cellular plasma membranes [69]). In our previous study, we showed that cholesterol affects the receptor structure, in the equilibrated part of the simulation. Therefore, to include the cholesterol-driven allosteric effects in the model, we used as starting conformation for hA_2A_R the last snapshot of the our previous 800-ns MD simulation of cholesterol-bound hA_2A_R with caffeine, embedded in a membrane with the same composition [27].

An educated guess of the ZMA binding pose in the cholesterol-bound hA_2A_R was obtained by superimposing ZMA via Pymol [70] software in the binding cavity, using as a template the configuration that ZMA has in the 3PWH X-ray structure [21]. Structural comparison (Figure S1) and Root Mean Square Deviation (RMSD) (Figure S2) across most of the X-ray structures of ZMA available so far in complex with hA_2A_R, is offered in the Supplementary Materials.

The protonation state of histidine residues, and in particular of H264 involved in forming the salt bridge with E169, was evaluated by PROPKA [71] and cross-checked within available hA_2A_R crystal structures (see Section S1). The AMBER99SB-ILDN force fields [72], the Slipids [73,74] and the TIP3P [75] force fields were used for the protein and ions, the lipids, and the water molecules respectively. The General Amber force field (GAFF) parameters [76] were used for ZMA, along with the RESP atomic charge using Gaussian 09 [77] with the HF-6-31G* basis set [78,79]. MD simulations were performed using Gromacs v4.5.5 package [80]. The total system is a 14.3 nm × 10.8 nm × 9.6 nm box, including 248 POPC lipids, 204 POPE lipids, and 141 cholesterol molecules. The total number of atoms in the system is 151,850. The computational protocols utilized in the previous study [27] was applied here for the MD simulation of ZMA/hA_2A_R complex. Specifically, MD simulation was conducted in the NPT ensemble (constant pressure and temperature) under periodic boundary conditions. Constant temperature and pressure conditions were achieved via independently coupling protein, lipids, solvent and ions to Nosè‒Hoover thermostat [81] at 310 K and Andersen‒Parrinello‒Rahman Barostat [82] at 1 atm. The Particle Mesh Ewald method [83] was used to treat the long-range electrostatic interaction with a real space cutoff of 1.2 nm. A 1.2-nm cutoff was used for the short-range non-bonded interaction. A time step of 2 fs was set. The LINCS algorithm [84] was applied to constrain all bonds involving hydrogen atoms. The final system was equilibrated for 20 ns under constant pressure and temperature (NPT ensemble) before metadynamics simulation.

### 4.2. Metadynamics Simulations 

The well-tempered metadynamics approach [17,18], an enhanced sampling algorithm within the framework of classical MD, was applied together with the computational protocol above to delineate the free energy profile for the binding of ZMA to hA_2A_R within the solvated neuronal-like membrane model (see Section S1 for further details on the methods). The deposition rate of the Gaussian bias terms was set to 1 ps and the initial height to 1.0 kJ/mol, with a bias factor of 15. To obtain the free energy profile of ZMA binding to hA_2A_R, we used two different collective variables, termed here CV_1_ and CV_2_ [19]. Specifically, CV_1_ was defined as the distance between the center of mass (COM) of ZMA and COM of Cα atoms of the transmembrane helical bundles of hA_2A_R along the membrane normal (Z-axis). CV_2_ corresponded to the distance between Cα atoms of H264 and E169. Gaussian widths of 0.05 nm were selected for CV_1_ and CV_2_, respectively, based on inspection of the initial dynamics of the system during equilibration. To restrict the sampling of conformational states in which the ligand was in contact with the protein, lower and upper limits of 1.5 nm and 3.8 nm, respectively, for the values of CV_1_ were enforced using steep harmonic potentials with an elastic constant of 250 kJ/nm^2^. Besides, one unbiased CV_3_ representing the XY component of the distance between the COM of ligand and the COM of Cα atoms of the transmembrane helical bundles of hA_2A_R was enforced below 1.2 nm so that the ligand would not diffuse to solvent regions that are far away from the receptor. All calculations used the Gromacs 4.5.5 program with the Plumed 1.3 plugin [85]. The unbinding free-energy was calculated as in [86,87,88]. The contribution to the free energy of binding from the metadynamics $∆G^{MetaD}$ was calculated as the free energy difference between the local minimum A (CV_1_ = [1.58 nm, 1.75 nm], CV_2_ = [0.82 nm, 0.91 nm]) and the unbound state G (CV_1_ = [4.40 nm, 4.50 nm], CV_2_ = [1.10 nm, 1.30 nm]); see Appendix A for further details of G definition. The contribution for CV_1_ > 4.5 nm ($∆G^{Elec}$) was estimated through the nonlinear Poisson‒Boltzmann equation by using APBS 1.4 program [34]. The setup for $∆G^{Elec}$ calculation is the following: The interior dielectric constant of the hA_2A_R was set to 4 and that of the solvents to 80. The concentration of sodium and chloride ions are set to 0.15 M. The total calculated value for the free energy of binding was obtained as $∆G=∆G^{MetaD}+∆G^{Elec}$. The standard-state free energy of binding was calculated by $∆G^{0}=∆G-RT\ln∆\left(\frac{\left[P\right]}{{\left[P\right]}^{0}}\right)$. R is the molar constant, [P] is the concentration of the protein in our simulation box, and ${\left[P\right]}^{0}$ = 1 M is the standard-state concentration [88]. $∆G^{0}$ was compared with the experimental binding free energies through the relationship $∆G^{0}=RTlnk_{eq}$, where $k_{eq}$ is the experimental equilibrium constant.

Volume analysis of the OBS of hA_2A_R was performed with trj_cavity 2.1 [89]. The residues comprising the OBS of hA_2A_R are defined as those within 0.6 nm of ZMA in the X-ray structure (PDBid:3PWH) [21]. Calculation of number of water molecules was performed with VMD [90]. The number of water molecules within 4 Å of ZMA is averaged over frames collected for each state. Coevolution analysis was performed with the web-based tool CoeViz [46] integrated in the web server POLYVIEW-2D [91].

### 4.3. Experimental Affinity Testing 

#### 4.3.1. Cell Culture

The cells were grown at 37 °C in 5% CO_2_/95% air adherently and kept in Ham’s F12 Nutrient Mixture, containing penicillin (100 U/mL), 10% fetal bovine serum, Geneticin (G418, 0.2 mg/mL), streptomycin (100 μg/mL), and l-glutamine (2 mM). Cells were split two or three times per week at a ratio between 1:5 and 1:20. The culture medium was removed and the cells were washed with PBS buffer (pH 7.4), scraped off, suspended in 1 mL PBS per dish, and stored at −80 °C, to prepare them for binding assays.

Membrane preparation for radioligand binding experiments. The cell were prepared as in [92]. The frozen cell suspension was thawed and homogenized on ice (Ultra-Turrax, 1 × 30 s at full speed). The homogenate was next centrifuged for 10 min (4 °C) at 600× *g.* The supernatant was then centrifuged for 60 min at 50,000× *g* after that, the membrane pellet was suspended again in 50 mM Tris/HCl buffer (pH 7.4) and frozen in liquid nitrogen at a protein concentration of 6 mg/mL. Finally, it was stored at −80 °C. Protein estimation used a naphthol blue black photometric assay [93] after solubilization in 15% NH_4_OH containing 2% SDS (*w*/*v*); human serum albumin served as a standard.

#### 4.3.2. Experimental Binding Affinity

Binding experiments used membranes from CHO K1 cells stably expressing the human A2A adenosine receptor. [3H]ZM 241385 (0.8 nM in competition experiments) as radioligand was used to obtain dissociation constant of [3H]ZM 241385 and the inhibition constant of not tritiated ZM 241385. Membrane homogenates with a protein content of 15 µg immobilized in a gel matrix were incubated with the radioligands in a total volume of 1500 µL 50 mM Tris/HCl buffer pH 7.4. This method produces the same results as conventional separation techniques and will be published in detail elsewhere. After an incubation time of 70 min the immobilized membrane homogenates were washed with water and transferred into scintillation cocktail (5 mL each, Ultima Gold, Perkin Elmer, Waltham, MA, USA). Liquid scintillation counter (Beckman Coulter, Brea, CA, USA) was used to measure the radioactivity of the samples (bound radioactivity). All binding data were calculated by non-linear curve fitting with a computer-aided curve-fitting program (Prism version 4.0, GraphPad Software, Inc., La Jolla, CA, USA).

## 5. Conclusions

Neuronal hA_2A_Rs, like other human GPCRs, are important pharmaceutical targets [94]. Here, we have presented a metadynamics study of the interaction between the high-affinity ligand ZMA and hA_2A_R, embedded in a solvated neuronal-like membrane environment. The calculations are consistent with the available experimental data and point to a clear and important role of lipids and of the second extracellular loop for ZMA’s molecular recognition process.

## Supplementary Materials

The following are available online, Section S1: Well-tempered Metadynamics, Section S2: H264^7.29^–E169^ECL2^ salt bridge: intramolecular interactions, Section S3: H264^7.29^ and E169^ECL2^ salt bridge: conservation across A2Ars, Section S4: Sodium allosteric binding site, Table S1: H264^7.29^ protonation state across hA_2A_R X-ray structures, Table S2: Ligand hydration (defined here as the number of water molecules within 4 Å of ZMA) and OBS volume for free energy minima A, B, C, D, E and F, Table S3. Conservation of residues composing the VBS of hA_2A_R, as emerging from our calculations, Table S4. Amino acid coevolution profile, Table S5. Presence of residue coevolution between orthosteric binding site (OBS) and extracellular loops (ECLs) of human receptors in class A, B, C and F, Figure S1: Receptor ligand interaction 2D scheme obtained by MOE (Molecular Operating Environment), Figure S2. Pairwise Root Main Square Deviation (RMSD) matrix, Figure S3. ZMA binding poses in the orthosteric binding site corresponding to minima B and C, Figure S4: Superimposition of hA_2_AR representative structure in the minima **B** (yellow tube) and **C** (cyan tube), Figure S5: Conservation of solvent-exposed motif of ECL2 in human Adenosine receptor subfamily, Figure S6: Conservation of solvent-exposed motif of ECL2 in Adenosine receptor A_2_R across different species, Figure S7: Conservation of H264^7.29^ and E169^ECL2^ in Adenosine receptor A_2A_R across different species, Figure S8: Conservation of H264^7.29^ and E169^ECL2^ in human Adenosine receptor subtypes hA_1_R, hA_2A_R, hA_2B_R, hA_3_R.

## Appendix A

The binding free energy depends on the difference between the free energy of fully bound state G**_B_** and that of the unbound state (G**_U_**) in which ZMA is located at infinite distance from the receptor:

∆G = G**_U_** − G**_B_**

However, this calculation of G**_U_** is not possible given the necessarily finite size of the simulation box. To circumvent this problem, let us rewrite ∆G as the sum of two contributions:

∆G = G**_U_** − G**_G_**+ G**_G_** − G**_B_** = G_WTM_ − ∆G_residual_

**G** is the relative minimum represented in Figure 3. G_WTM_ is by far the largest contribution, and it calculated by well-tempered metadynamics. G_residual_ is well approximated by the free energy associated with long-range electrostatic interactions with the protein (∆G_residual_ ≈ ∆G_electr_). Indeed, the ligand in **G** does not form direct H-bonds and/or hydrophobic contacts with the protein. It is at about 0.8 nm from the protein atoms, separated by water molecules. Hence, it forms only long-range electrostatic interactions with the membrane and the protein. However, the latter are vanishingly small because the ligand is at least 2.5 nm from the membrane. In addition, we notice that in **G** the conformational degrees of freedom of the ligand are not partially restricted. Hence, the entropic contribution associated with these degrees of freedom is expected to be also vanishingly small.

∆G_electr_ is expected to be much smaller than ∆G_WTM_, as ∆G_electr_ values lower than 2k_B_T have been calculated for other ligand/protein interactions [88,95]. Indeed, a posteriori, ∆G_electr_, as calculated using the APBS 1.4 program [34], turns out to be only 0.95 Kcal/mol. We conclude that the corrections due to the finite size of the simulation box are small. In other words, the ligand in **G** interacts very weakly with the protein. Hence, the errors in the calculations of this term are not expected to dramatically affect the binding free energy.
