# Supplementary material for: Role of Extracellular Loops and Membrane Lipids for Ligand Recognition in the Neuronal Adenosine Receptor Type 2A: An Enhanced Sampling Simulation Study
Source: Molecules. 2018 Oct 12;23(10):2616. doi: 10.3390/molecules23102616 (PMC6222423; doi:10.3390/molecules23102616)
Supplement: Supplementary file 1 [file molecules-23-02616-s001.pdf]

molecules

MDPI

Article–Supporting Information

Role of Extracellular Loops and Membrane Lipids for Ligand Recognition in the Neuronal Adenosine Receptor Type 2A: An Enhanced Sampling Simulation Study

Ruyin Cao [1], Alejandro Giorgetti [1,2], Andreas Bauer [3], Bernd Neumaier [4], Giulia Rossetti [1,5,6,*] and Paolo Carloni [1,7,8,9,*]

1 Institute of Neuroscience and Medicine (INM-9) and Institute for Advanced Simulation (IAS-5), Forschungszentrum Jülich, Wilhelm-Johnen-Strasse, 52425 Jülich, Germany; caobb0214@gmail.com (R.C.); alejandro.giorgetti@univr.it (A.G.)
2 Department of Biotechnology, University of Verona, Strada Le Grazie 15, 37134 Verona, Italy
3 Institute for Neuroscience and Medicine (INM)-2, Forschungszentrum Jülich, 52428 Jülich, Germany; an.bauer@fz-juelich.de
4 Institute for Neuroscience and Medicine (INM)-5, Forschungszentrum Jülich, 52428 Jülich, Germany; b.neumaier@fz-juelich.de
5 Jülich Supercomputing Center (JSC), Forschungszentrum Jülich, 52428 Jülich, Germany
6 Department of Oncology, Hematology and Stem Cell Transplantation, University Hospital Aachen, 52078 Aachen, Germany
7 Department of Physics, RWTH Aachen University, 52078 Aachen, Germany
8 Institute for Neuroscience and Medicine (INM)-11, Forschungszentrum Jülich, 52428 Jülich, Germany;
9 Department of neurology, University Hospital Aachen, 52078 Aachen, Germany
* Correspondence: g.rossetti@fz-juelich.de (G.R.); p.carloni@fz-juelich.de (P.C.); Tel.: +49-2461-61-8933 (G.R.); +49-2461-61-8942 (P.C.); Fax: +49-2461-61-4823 (G.R.); +49-2461-61-4823(P.C.)

SUPPORTING INFORMATION

Section S1: Well-tempered Metadynamics

Well-tempered metadynamics (WTM) [1,2] works by introducing of a history-dependent potential V acting on a selected number of slow degrees of freedom, the collective variables (CVs). This forces the dynamics to explore conformations that were not previously visited and discourages the system from returning to these regions. Therefore, it allows the system escaping minima along low free energy paths and exploring other minima in the free energy landscape. This occurs independently of the minima one starts from. From the potential V, one can calculate the free energy.

Let us consider only one CV (*s* hereafter) for simplicity. The free energy surface along *s*, F(s), can be then calculated from the equation:

$$V(s, t \to \infty) = -\frac{T + \Delta T}{\Delta T} F(s)$$

In the well-tempered formulation[1] (the ones implemented here), the potential used to bias the dynamics in order to accelerate the sampling is:

$$V(s, t) = \Delta T \ln\left(1 + \frac{\omega N(s, t)}{\Delta T}\right)$$

where ω has the dimension of an energy rate, ΔT is a temperature and N(s, t) comes from the biased simulation. As pointed out by the authors [1], an important property of this formulation is that, it insures the bias eventually to converge, yet "slow enough for the final result not to depend on the initial condition V (s, 0)."

Section S2: H264$^{7.29}$- E169^{ECL2} salt bridge: intramolecular interactions.

H264$^{7.29}$ is predicted to be doubly protonated by PROPKA[3] at neutral pH. Hence, it is expected to form a salt-bridge with E169^{ECL2}. 18 out of 19 X-ray hA$_{2A}$R/ligand structures at pH<7.6 feature this putative salt bridge (see Table S1). The only exception occurs with the agonist UK43907 (PDBid: 3QAK). Here, because of steric reasons, the bulky ring of UK43907 causes a displacement of H264$^{7.29}$ and E169^{ECL2}, with an increase of 3~4 Å of the Cα-Cα distance).[1] The salt bridge might behave as a 'gate' closing the ligand in the inner binding site and separating it from the vestibular binding site[4-6].

Section S3: H264$^{7.29}$ and E169^{ECL2}salt bridge: conservation across A2ARs.

The following analysis shows that the two groups are fairly conserved across all A2ARs, while this is not the case for all the other human adenosine receptors.

Methods. By using the CLUSTAL Omega (1.2.2) web server[7], we investigated all A$_{2A}$Rs, along with the other three adenosine receptors (A$_1$Rs, A3Rs and A$_{2B}$Rs) deposited in the UniProt **[8]** UniProtKB database (http://www.uniprot.org/). The conservation of E169^{ECL2} and H264$^{7.29}$ across all A$_{2A}$Rs are 61 % and 39 %, respectively.

Results. H264$^{7.29}$ is replaced by R and V for the 33% and 17% of the cases, respectively (Figure S7). In the R264 variants, the residues in position 169 are always E. Hence, the putative salt bridge (either formed by E169 and H264, or by E169 and R264) is conserved for slightly more than 60% across all A$_{2A}$Rs.

The pairwise sequence identities of the other human adenosine receptors (hA$_1$R, hA$_{2B}$R, and hA$_3$R) with A$_{2A}$R ranges from 58% to 39% (Figure S8). Position 169 is E hA$_1$R and hA$_{2B}$R while it is V in hA$_3$R (see Figure S7). Position 264 is H in hA$_1$R, E in hA$_3$R, N in hA$_{2B}$R. Here, N is embedded between two K's in a "KNK" motif.

The conservation of the E169^{ECL2}-H264$^{7.29}$ positions across all A$_1$Rs is 58% (Figure S9). E169 and H264$^{7.29}$ are conserved for 92% and 33.3% of the cases, respectively. The position 264 features also both positively (26%) and negatively (33%) charged residues.

The E169^{ECL2}-H264$^{7.29}$ positions are not conserved across all A$_{2B}$Rs and A$_3$Rs (Figure S10-S11). It may be replaced by residues which could form H-bond interactions (as opposed to a salt bridge) or a salt bridge other than that formed here: indeed, in the case ofA$_3$Rs, the E169/K264 pair is present in 30% of the sequences.

Section S4: Sodium allosteric binding site.

This site has a fundamental importance in allosteric modulation of GPCRs [9,10]. The residues that mostly contribute to sodium binding along the GPCRs family, i.e S$^{3.39}$, N$^{7.45}$ and D$^{2.50}$ are described in literature as highly conserved. Position 2.50, in particular is an aspartic acid in 90% of the eukaryotic GPCRs, accordingly to our alignments taken from a curated multiple sequence alignment from GPCRdb. This residue can highly modulate the function of GPCRs. Indeed, the role of sodium modulation is well known for several GPCRs [10]. Mutagenesis studies on residues involved in the sodium ion coordination, and in particular D$^{2.50}$, highlighted the different effects that allosteric sodium may have in various class A GPCRs signaling [9]. Indeed, D$^{2.50}$ replacement with uncharged amino acids can drastically reduce the agonist-induced G protein activation [11,12] [13] [14,15] or modulate the allosteric effect of the G-protein on ligand binding [16]. The presence of sodium ions in the allosteric cavity can also exert different effects on the constitutive signaling of GPCRs [17,18]. In many cases, the presence of bound sodium seems to stabilize the inactive conformation of the receptor reducing the constitutive G-protein [13,15], whereas in other receptors

---

[1] In high pH environment, H264$^{7.29}$ is instead predicted as deprotonated, and the salt bridge with E169^{ECL2} is expected to be broken. This is indeed the case for the remaining 5 structures crystalized at pH > 7.6 (see Table S1).

the substitution of sodium coordinating $D^{2.50}$ abolishes the constitutive G-protein coupling and activation without affecting the agonist-stimulated activity [19]. Exhaustive studies have also revealed that the sodium pocket collapses due to the activation-related movements of the transmembrane helixes [18]. Recently, we have shown that the disruption of the sodium binding site of GPR3 strongly biased the receptor to the inactive state [20]. Thus, most of the studies agree with the fact that the constitutive activity can be dramatically affected by mutations in this cavity. Indeed, a constitutive active mutant (CAM) on human mu-opioid receptors, has been observed to disrupt the allosteric sodium binding cavity, favoring the exploration of active-like conformations even in the apo state [21]. In particular, for A2A receptors, very recently, White and collaborators [22] obtained the crystal structures of agonist complexes for two variants sodium binding site, D 52 $^{2.50}$N and $S91^{3.39}$A. In both cases the structures are active-like but, the variants induce important changes in the activation motif NPxxY. The authors, combining several experimental techniques provide a basis for understanding the role of the sodium-coordinating residues on stability and G-protein signaling.

4. Tables

Table S1. $H264^{7.29}$ protonation state across $hA_{2A}R$ X-ray structures. The table reports: the resolution of the X-ray structures, the type of ligand (antagonists and agonists are colored in red or blue respectively), the pH of crystallization, the protonation state of $H264^{7.29}$ at corresponding crystallization pH value ($H264^{7.29}$ protonation), as predicted using PROPKA[3], the presence of $H264^{7.29}$ and $E169^{ECL2}$ salt-bridge/HB interactions and $H264^{7.29}$ CA - $E169^{ECL2}$ CA distances (Å).

PDBid	Resolution	ligand	pH	$H264^{7.29}$ Protonation	$H264^{7.29}$ -$E169^{ECL2}$ salt bridge—/HB interaction	CA-CA distance	Reference
3EML	2.6	ZMA	6.5	Yes	Yes	11.5	[23]
4EIY	1.8	ZMA	5.0	Yes	Yes	11.4	[24]
3VG9	2.7	ZMA	6.5	No	Yes	11.3	[25]
3VGA	3.1	ZMA	6.5	Yes	Yes	11.0	[25]
2YDO	3.0	adenosine	7.6	Yes	Yes	11.5	[26]
2YDV	2.6	NECA	6.4	Yes	Yes	11.5	[26]
3QAK	2.7	UK-432097	5.0	Yes	No	15.3	[27]
4UHR	2.6	CGS21680	4.8	Yes	Yes	12.1	[28]
4UG2	2.6	CGS21680	4.8	Yes	Yes	11.2	[28]
3PWH	3.3	ZMA	8.1	No	No	11.9	[29]

3RFM	3.6	caffeine	8.2	No	No	11.8	[29]
3REY	3.3	xanthine	8.2	No	No	11.9	[29]
3UZC	3.3	T4E	8.0	No	No	11.7	[30]
3UZA	3.3	T4G	8.0	No	No	11.8	[30]
5G53	3.4	NECA	5.5	Yes	Yes	11.1	[31]
5IU4	1.7	ZMA	5.5	Yes	Yes	11.4	[5]
5IU7	1.9	6DY	5.4	Yes	Yes	11.4	[5]
5IU8	2.0	18F	5.5	Yes	Yes	11.4	[5]
5IUA	2.2	6DX	5.4	Yes	Yes	11.4	[5]
5IUB	2.1	6DV	5.5	Yes	Yes	11.4	[5]
5K2A	2.5	ZMA	5.0	Yes	Yes	11.4	[32]
5K2B	2.5	ZMA	5.0	Yes	Yes	11.4	[32]
5K2C	1.9	ZMA	5.0	Yes	Yes	11.4	[32]
5K2D	1.9	ZMA	5.0	Yes	Yes	11.4	[32]
5UIG	3.5	8D1	6.5	Yes	Yes	10.5	[33]
5UVI	3.2	ZMA	5.0	Yes	Yes	11.3	[34]
5JTB	2.8	ZMA	5.2	Yes	No	11.5	[35]
5N2R	2.8	8JN	5.5	Yes	No	12.2	[36]
5MZP	2.1	Caffeine	5.5	Yes	Yes	11.5	[36]
5MZJ	2.0	TEP	5.5	Yes	Yes	11.3	[36]
5NM4	1.7	ZMA	5.0	Yes	Yes	11.3	[37]
5NM2	1.9	ZMA	5.0	Yes	No	12.9	[37]
5NLX	2.1	ZMA	5.0	Yes	Yes	11.4	[37]
5VRA	2.4	ZMA	5.0	Yes	Yes	11.5	[38]

6AQF	2.5	ZMA	5.0	Yes	Yes	11.5	[39]
5OM4	2.0	T4E	5.3	Yes	Yes	11.4	[40]
5OM1	2.1	T4E	5.3	Yes	Yes	11.5	[40]
5OLZ	1.9	T4E	5.3	Yes	Yes	10.3	[40]
5OLV	2.0	LUAA47070	5.3	Yes	Yes	11.5	[40]
5OLO	3.1	Tozadenant	5.3	Yes	No	12.4	[40]
5OLK	2.6	Vipadenant	5.3	Yes	Yes	11.4	[40]
5OLG	1.9	ZMA	5.3	Yes	Yes	11.4	[40]
5WF6	2.9	UKA	5.0	Yes	No	14.6	[22]

Table S2. Ligand hydration (defined here as the number of water molecules within 4 Å of ZMA) and OBS volume for free energy minima **A**, **B**, **C**, **D**, **E** and **F**. .

State	OBS Volume (nm^3)	Ligand hydration
A	0.38±0.07	12±3
B	0.42±0.07	10±3
C	0.45±0.08	13±3
D	0.34±0.06	21±4
E	0.39±0.06	16±4
F	0.33±0.06	33±6

Table S3. Conservation of residues forming the VBS of $hA_{2A}R$, as emerging from our calculations. Sequences of four human adenosine subtypes and 18 sequences of adenosine receptor type 2A across species were used for multiple sequence alignment on the web server of CLUSTAL O (1.2.2) [7].

Residue	Conservation in human ARs	Conservation in $A_{2A}Rs$
$M1^{1.27}$	0%	33%
$P2^{1.28}$	0%	33%
$Y9^{1.35}$	100%	50%
$E13^{1.39}$	100%	50%

S67$^{2.65}$	75%	50%
T68$^{2.66}$	100%	50%
G69$^{2.67}$	100%	50%
N154^{ECL2}	0%	39%
H155^{ECL2}	0%	11%
A165^{ECL2}	0%	17%
M270$^{7.35}$	50%	72%
Y271$^{7.36}$	75%	44%

Table S4. Amino acid coevolution profile computed using the Coeviz tool within the web server polyview-2d [41]. The chi-squared covariance, weighted by phylogeny derived from alignments of hA$_{2A}$R sequence (as defined in PDBid 3PWH [29]) against NCBI NR database with 90% identity [41]. For each amino acid, the genetic number and binding site location are annotated.

	**VBS**	**VBS**	**VBS**	**VBS**	**VBS**	**OBS**	**OBS**	**VBS'**	**VBS'**	**OBS**	**OBS**	**OBS/VBS**	**OBS/VBS**	**OBS**	**OBS**
	Y9$^{1.35}$	E13$^{1.39}$	I64$^{2.61}$	S67$^{2.64}$	G69^{ECL1}	V84$^{3.32}$	L85$^{3.33}$	G142^{ECL2}	W143^{ECL2}	M177$^{5.40}$	N181$^{5.43}$	M270$^{7.34}$	Y271$^{7.35}$	I274$^{7.38}$	H278$^{7.42}$
Y9$^{1.35}$	0.133	0.551	0.349	0.323	0.354	0.329	0.456	0.413	0.419	0.445	0.444	0.389	0.272	0.462	0.522
E13$^{1.39}$		0.062	0.431	0.314	0.426	0.471	0.512	0.522	0.499	0.562	0.532	0.455	0.328	0.573	0.652
I64$^{2.61}$			0.097	0.184	0.206	0.219	0.295	0.262	0.279	0.306	0.279	0.267	0.206	0.350	0.404
S67$^{2.64}$				0.142	0.104	0.207	0.346	0.175	0.187	0.249	0.198	0.238	0.126	0.320	0.304
G69^{ECL1}					0.172	0.302	0.293	0.282	0.287	0.358	0.329	0.272	0.203	0.351	0.434

V84$^{3.32}$						0.047	0.399	0.329	0.336	0.417	0.462	0.438	0.311	0.368	0.460
L85$^{3.33}$							0.069	0.356	0.363	0.463	0.391	0.431	0.345	0.401	0.495
G142^{ECL2}								0.132	0.639	0.436	0.364	0.312	0.214	0.410	0.498
W143^{ECL2}									0.135	0.442	0.370	0.319	0.223	0.405	0.502
M177$^{5.40}$										0.107	0.467	0.385	0.321	0.486	0.549
N181$^{5.43}$											0.073	0.418	0.273	0.420	0.510
M270$^{7.34}$												0.047	0.307	0.372	0.441
Y271$^{7.35}$													0.057	0.286	0.322
I274$^{7.38}$														0.122	0.557
H278$^{7.42}$															0.062

Table S5. Presence of residue coevolution between orthosteric binding site (OBS) and extracellular loops (ECLs) of human receptors in class A, B, C and F. X-ray structures of 27 human GPCRs with OBS-bound ligand were used for evolutionary correlation analysis with Coeviz tool in polyview-2d webserver **[41]**. The threshold of PCS score was chosen as >0.3 to identify evolutionarily correlated residue pairs. Among them, 22 GPCRs have residue-based ECL-OBS coevolution relation. **'0"** ECL is missing in X-ray structure; "Yes" presence of residue-based evolutionary correlation; "No" absence of residue-based evolutionary correlation.

Receptor	PDBid	Class	ECL1-OBS Correlation	ECL2-OBS Correlation	ECL3-OBS Correlation
β2-adrenergic receptor	3D4S	A	No	Yes	Yes
CXCR4	3ODU	A	Yes	Yes	Yes
D3 receptor	3PBL	A	Yes	No	Yes
A2A receptor	3PWH	A	Yes	Yes	Yes
H1 receptor	3RZE	A	Yes	Yes	Yes
M1 receptor	5CXV	A	Yes	Yes	Yes
S1P1 receptor	3V2Y	A	Yes	Yes	Yes
κ receptor	4DJH	A	Yes	Yes	0
NOP receptor	4EA3	A	No	No	No
PAR1 receptor	3VW7	A	Yes	Yes	Yes
5-HT1B receptor	4IAR	A	Yes	No	0
5-HT2B receptor	4IB4	A	Yes	No	No
SMO receptor	4JKV	F	Yes	Yes	Yes
CRF1 receptor	4K5Y	B	Yes	Yes	0
glucagon receptor	4L6R	B	0	No	No
CCR5 receptor	4MBS	A	No	No	No
M2 receptor	3UON	A	Yes	Yes	Yes
δ receptor	4N6H	A	No	No	No
mGlu1 receptor	4OR2	C	Yes	Yes	No
mGlu5 receptor	5CGD	C	Yes	Yes	Yes
P2Y12 receptor	4NTJ	A	No	No	Yes

FFA1 receptor	4PHU	A	Yes	Yes	Yes
OX2 receptor	4S0V	A	Yes	Yes	Yes
AT1 receptor	4YAY	A	No	No	No
LPA1 receptor	4Z35	A	Yes	Yes	Yes
OX1 receptor	4ZJC	A	Yes	Yes	No
M4 receptor	5DSG	A	Yes	Yes	Yes

5. Figures

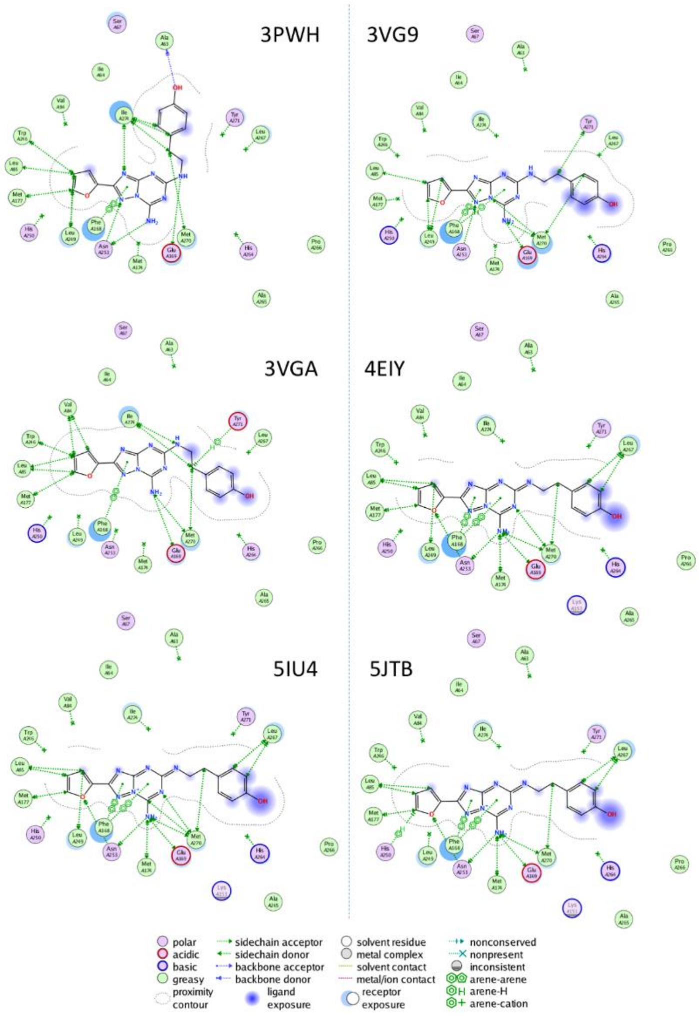

Figure S1-part A. Receptor ligand interaction 2D scheme obtained by MOE (Molecular Operating Environment) [42].

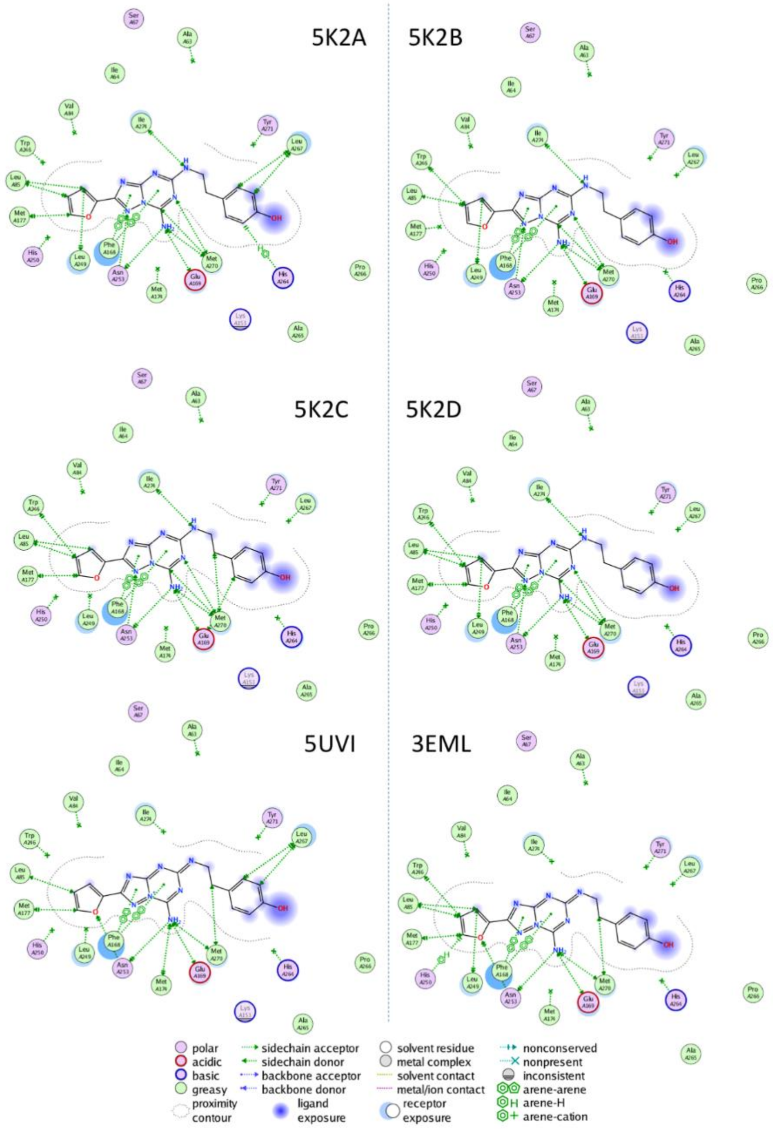

Figure S1-part B. Receptor ligand interactions 2D scheme obtained by MOE (Molecular Operating Environment) [42].

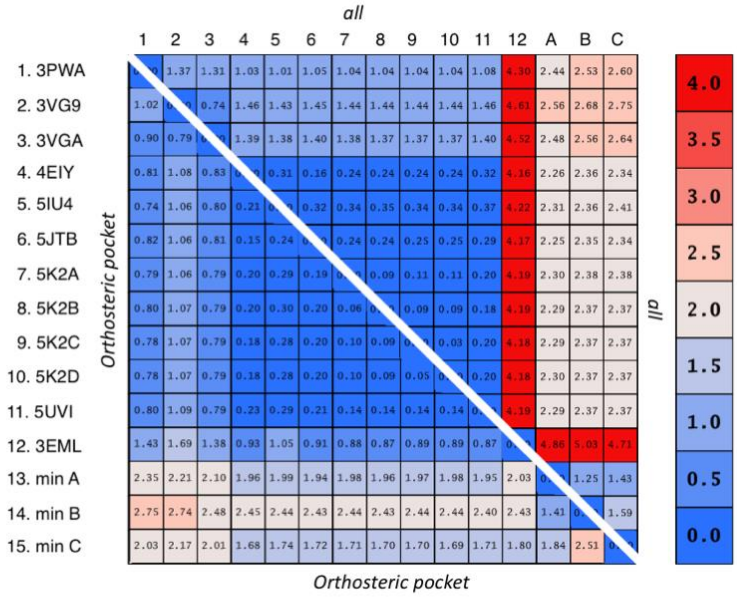

Figure S2. Pairwise Root Main Square Deviation (RMSD) matrix across twelve of the deposited structures of ZMA-bound $hA_{2A}R$ receptor and the minima **A**, **B**, **C** calculated in Å considering the Cα atoms of the overall receptor (upper-right triangle) or the residues belonging to the binding site (lower-left triangle).

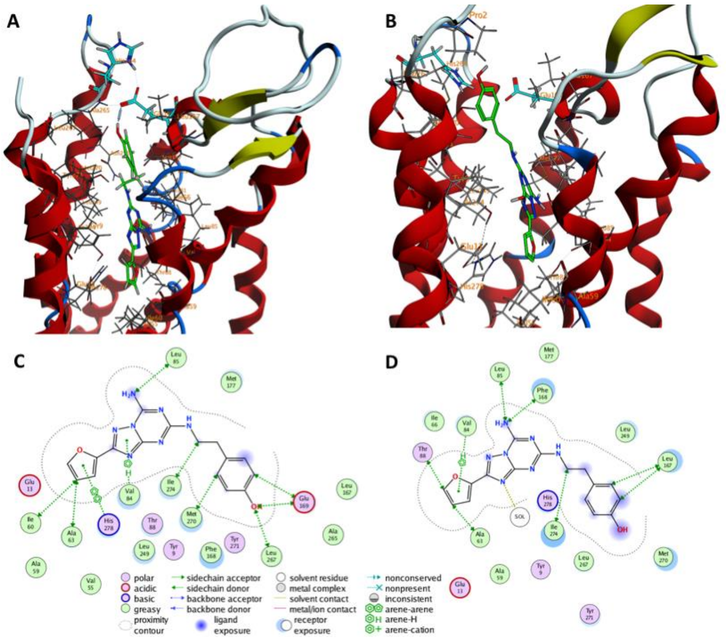

Figure S3. ZMA binding poses in the orthosteric site corresponding to minima **B** and **C** in Figure 1 are shown in A and B panels, respectively, as 3D representation. The protein backbone is rendered as cartoon, ZMA is shown as a green licorice, residues interacting with ZMA are shown as grey lines. The E169^{ECL2} and H264$^{7.29}$ residues are shown in cyan licorice. Hydrogen, oxygen and nitrogen atoms are specifically colored in white, red and light blue, respectively. C-D) 2D scheme of binding poses in A) and B), respectively.

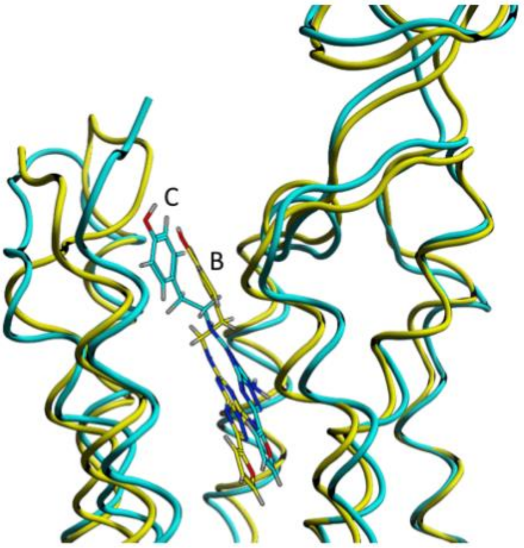

Figure S4 Superimposition of hA_2AR representative structure in the minima **B** (yellow tube) and **C** (cyan tube). ZMA is shown in yellow and cyan licorice representation for **B** and **C** minima, respectively.

```
sp|P0DMS8|AA3R_HUMAN     VTTHRRIWLALGLCWLVSFLVGLTPMFGWNMKLTSEYH--------------RNVTFLSCQ
sp|P30542|AA1R_HUMAN     VVTPRRAAVAIAGCWILSFVVGLTPMFGWNNLSAVER----AWA---ANGSMGEPVIKCE
sp|P29274|AA2AR_HUMAN    LVTGTRAKGIIAICWVLSFAIGLTPMLGWNN-------CGQPKEGKNHSQGCGEGQVACL
sp|P29275|AA2BR_HUMAN    LVTGTRARGVIAVLWVLAFGIGLTPFLGWNSKDSATNNCTEPWDGTTNESCC---LVKCL
                         :.*  *    :.   *:::* :****::***                          : *
```

Figure S5. Conservation of solvent-exposed motif of ECL2 in human Adenosine receptor subfamily. This multiple sequence alignment was generated using the web server of CLUSTAL O (1.2.2) [7]. Amino acid residues are colored according to this scheme: small and hydrophobic residues including aromatic residues are colored in red, acidic residues are colored in blue, basic residues are colored in magenta, and hydroxyl, sulfhydryl, amine residues and glycine are colored in green. Same alignment method and coloring schemes are applied in the following Figures S6-11.

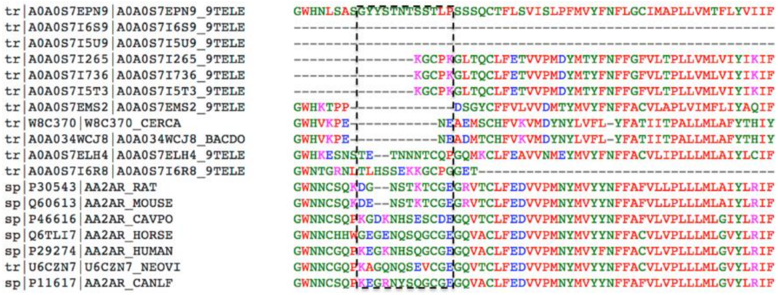

Figure S6. Conservation of solvent-exposed motif of ECL2 in Adenosine receptor A2R across different species. Color-code and alignment method as in Figure S5.

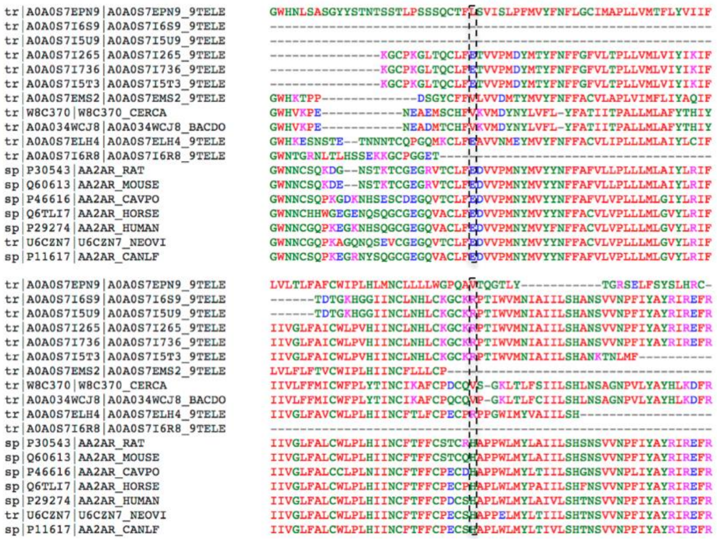

Figure S7. Conservation of H264$^{7.29}$ and E169^{ECL2} in Adenosine receptor A$_{2A}$R across different species. Color-code and alignment method as in Figure S5.

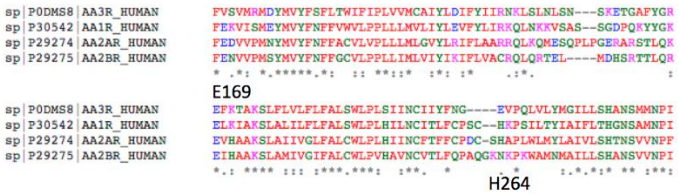

Figure S8. Conservation of H264$^{7.29}$ and E169^{ECL2} in human Adenosine receptor subtypes hA$_1$R, hA$_{2A}$R, hA$_{2B}$R, hA$_3$R. Color-code and alignment method as in Figure S5.

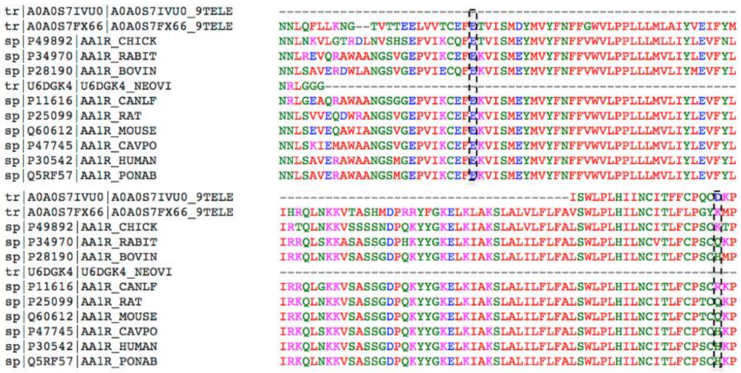

Figure S9. Conservation of H264$^{7.29}$ and E169^{ECL2} in Adenosine receptor A$_1$R across different species. Color-code and alignment method as in Figure S5.

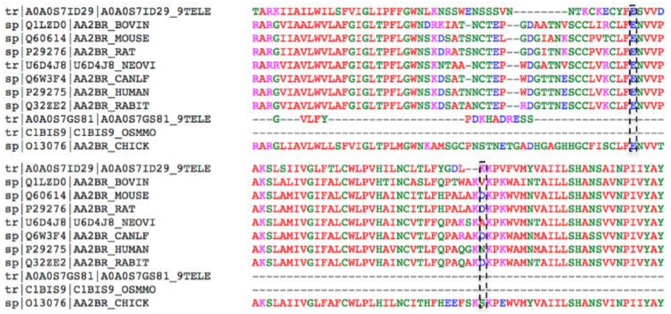

Figure S10. Conservation of H264$^{7.29}$ and E169^{ECL2} in Adenosine receptor A$_{2B}$R across different species. Color-code and alignment method as in Figure S5.

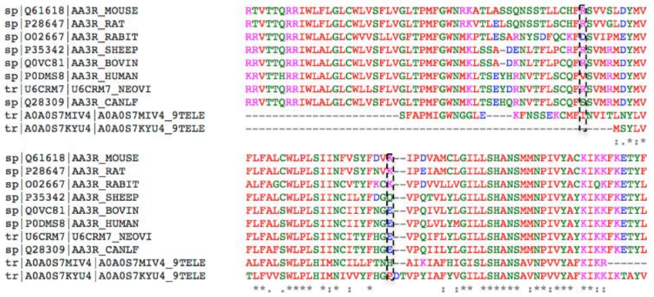

Figure S11. Conservation of H264$^{7.29}$ and E169^{ECL2} in Adenosine receptor A$_3$R across different species. Color-code and alignment method as in Figure S5.

References

1. Barducci, A.; Bussi, G.; Parrinello, M. Well-tempered metadynamics: a smoothly converging and tunable free-energy method. *Phys Rev Lett* **2008**, *100*, 020603, doi:10.1103/PhysRevLett.100.020603.
2. Laio, A.; Parrinello, M. Escaping free-energy minima. *Proc Natl Acad Sci U S A* **2002**, *99*, 12562-12566, doi:10.1073/pnas.202427399.
3. Dolinsky, T.J.; Czodrowski, P.; Li, H.; Nielsen, J.E.; Jensen, J.H.; Klebe, G.; Baker, N.A. PDB2PQR: expanding and upgrading automated preparation of biomolecular structures for molecular simulations. *Nucleic acids research* **2007**, *35*, W522-W525.
4. Dror, R.O.; Pan, A.C.; Arlow, D.H.; Borhani, D.W.; Maragakis, P.; Shan, Y.; Xu, H.; Shaw, D.E. Pathway and mechanism of drug binding to G-protein-coupled receptors. *Proc Natl Acad Sci U S A* **2011**, *108*, 13118-13123, doi:10.1073/pnas.1104614108.
5. Segala, E.; Guo, D.; Cheng, R.K.; Bortolato, A.; Deflorian, F.; Doré, A.S.; Errey, J.C.; Heitman, L.H.; IJzerman, A.P.; Marshall, F.H. Controlling the Dissociation of Ligands from the Adenosine A2A Receptor through Modulation of Salt Bridge Strength. *J Med Chem* **2016**, *59*, 6470-6479, doi:0.1021/acs.jmedchem.6b00653.

6. Yu, A.; Alberstein, R.; Thomas, A.; Zimmet, A.; Grey, R.; Mayer, M.L.; Lau, A.Y. Molecular lock regulates binding of glycine to a primitive NMDA receptor. *Proc. Natl. Acad. Sci. U.S.A.* **2016**, *113*, E6786-E6795, doi:10.1073/pnas.1607010113.
7. Sievers, F.; Wilm, A.; Dineen, D.; Gibson, T.J.; Karplus, K.; Li, W.; Lopez, R.; McWilliam, H.; Remmert, M.; Söding, J. Fast, scalable generation of high-quality protein multiple sequence alignments using Clustal Omega. *Mol Syst Biol* **2011**, *7*, 539, doi:10.1038/msb.2011.75.
8. Apweiler, R.; Bairoch, A.; Wu, C.H.; Barker, W.C.; Boeckmann, B.; Ferro, S.; Gasteiger, E.; Huang, H.; Lopez, R.; Magrane, M., et al. UniProt: the Universal Protein knowledgebase. *Nucleic Acids Res* **2004**, *32*, D115-119, doi:10.1093/nar/gkh131.
9. Katritch, V.; Fenalti, G.; Abola, E.E.; Roth, B.L.; Cherezov, V.; Stevens, R.C. Allosteric sodium in class A GPCR signaling. *Trends Biochem Sci* **2014**, *39*, 233-244, doi:10.1016/j.tibs.2014.03.002.
10. Liu, W.; Chun, E.; Thompson, A.A.; Chubukov, P.; Xu, F.; Katritch, V.; Han, G.W.; Roth, C.B.; Heitman, L.H.; Ijzerman, A.P., et al. Structural Basis for Allosteric Regulation of GPCRs by Sodium Ions. *Science* **2012**, *337*, 232-236, doi:papers3://publication/doi/10.1126/science.1219218.
11. Bihoreau, C.; Monnot, C.; Davies, E.; Teutsch, B.; Bernstein, K.E.; Corvol, P.; Clauser, E. Mutation of Asp74 of the rat angiotensin II receptor confers changes in antagonist affinities and abolishes G-protein coupling. *Proc Natl Acad Sci U S A* **1993**, *90*, 5133-5137, doi:papers3://publication/doi/10.1073/pnas.90.11.5133.
12. Perlman, J.H.; Colson, A.O.; Wang, W.; Bence, K.; Osman, R.; Gershengorn, M.C. Interactions between conserved residues in transmembrane helices 1, 2, and 7 of the thyrotropin-releasing hormone receptor. *J Biol Chem* **1997**, *272*, 11937-11942, doi:DOI 10.1074/jbc.272.18.11937.
13. Rose, P.M.; Krystek, S.R.; Patel, P.S.; Liu, E.C.; Lynch, J.S.; Lach, D.A.; Fisher, S.M.; Webb, M.L. Aspartate mutation distinguishes ETA but not ETB receptor subtype-selective ligand binding while abolishing phospholipase C activation in both receptors. *FEBS Lett* **1995**, *361*, 243-249, doi:papers3://publication/uuid/471942BF-DAEF-4806-8A6D-73B9F6B914F9.
14. Wang, C.D.; Gallaher, T.K.; Shih, J.C. Site-directed mutagenesis of the serotonin 5-hydroxytrypamine2 receptor: identification of amino acids necessary for ligand binding and receptor activation. *Mol Pharmacol* **1993**, *43*, 931-940, doi:papers3://publication/uuid/A250F338-5EDE-4CA9-BEC6-B0216DCD9F05.
15. Tao, Q.; Abood, M.E. Mutation of a highly conserved aspartate residue in the second transmembrane domain of the cannabinoid receptors, CB1 and CB2, disrupts G-protein coupling. *J Pharmacol Exp Ther* **1998**, *285*, 651-658, doi:papers3://publication/uuid/ED748195-132D-4CE9-8ECD-4286B8C312FD.
16. Li, B.; Nowak, N.M.; Kim, S.-K.; Jacobson, K.A.; Bagheri, A.; Schmidt, C.; Wess, J. Random Mutagenesis of the M3Muscarinic Acetylcholine Receptor Expressed in Yeast. *Journal of Biological Chemistry* **2005**, *280*, 5664-5675, doi:10.1074/jbc.M411623200.
17. Quitterer, U.; AbdAlla, S.; Jarnagin, K.; Muller-Esterl, W. Na+ ions binding to the bradykinin B2 receptor suppress agonist-independent receptor activation. *Biochemistry* **1996**, *35*, 13368-13377, doi:10.1021/bi961163w.
18. Seifert, R.; Wenzel-Seifert, K. Unmasking different constitutive activity of four chemoattractant receptors using Na+ as universal stabilizer of the inactive (R) state. *Receptor Channel* **2001**, *7*, 357-369, doi:papers3://publication/uuid/FB565E6D-18A6-4BC8-A885-5B240B755CEB.
19. Nie, J.J.; Lewis, D.L. Structural domains of the CB1 cannabinoid receptor that contribute to constitutive activity and G-protein sequestration. *J Neurosci* **2001**, *21*, 8758-8764, doi:Doi 10.1523/Jneurosci.21-22-08758.2001.
20. Capaldi, S.; Suku, E.; Antolini, M.; Di Giacobbe, M.; Giorgetti, A.; Buffelli, M. Allosteric sodium binding cavity in GPR3: a novel player in modulation of Abeta production. *Sci Rep-Uk* **2018**, *8*, 11102, doi:10.1038/s41598-018-29475-7.
21. Sena, D.M., Jr.; Cong, X.; Giorgetti, A.; Kless, A.; Carloni, P. Structural heterogeneity of the mu-opioid receptor's conformational ensemble in the apo state. *Sci Rep-Uk* **2017**, *8*, 45761, doi:10.1038/srep45761.
22. White, K.L.; Eddy, M.T.; Gao, Z.G.; Han, G.W.; Lian, T.; Deary, A.; Patel, N.; Jacobson, K.A.; Katritch, V.; Stevens, R.C. Structural Connection between Activation Microswitch and Allosteric Sodium Site in GPCR Signaling. *Structure* **2018**, *26*, 259-+, doi:10.1016/j.str.2017.12.013.
23. Jaakola, V.P.; Griffith, M.T.; Hanson, M.A.; Cherezov, V.; Chien, E.Y.; Lane, J.R.; Ijzerman, A.P.; Stevens, R.C. The 2.6 angstrom crystal structure of a human A2A adenosine receptor bound to an antagonist. *Science* **2008**, *322*, 1211-1217, doi:10.1126/science.1164772.
24. Liu, W.; Chun, E.; Thompson, A.A.; Chubukov, P.; Xu, F.; Katritch, V.; Han, G.W.; Roth, C.B.; Heitman, L.H.; AP, I.J., et al. Structural basis for allosteric regulation of GPCRs by sodium ions. *Science* **2012**, *337*, 232-236, doi:10.1126/science.1219218.

25. Hino, T.; Arakawa, T.; Iwanari, H.; Yurugi-Kobayashi, T.; Ikeda-Suno, C.; Nakada-Nakura, Y.; Kusano-Arai, O.; Weyand, S.; Shimamura, T.; Nomura, N., et al. G-protein-coupled receptor inactivation by an allosteric inverse-agonist antibody. *Nature* **2012**, *482*, 237-240, doi:10.1038/nature10750.
26. Lebon, G.; Warne, T.; Edwards, P.C.; Bennett, K.; Langmead, C.J.; Leslie, A.G.; Tate, C.G. Agonist-bound adenosine A2A receptor structures reveal common features of GPCR activation. *Nature* **2011**, *474*, 521-525, doi:10.1038/nature10136.
27. Xu, F.; Wu, H.; Katritch, V.; Han, G.W.; Jacobson, K.A.; Gao, Z.G.; Cherezov, V.; Stevens, R.C. Structure of an agonist-bound human A2A adenosine receptor. *Science* **2011**, *332*, 322-327, doi:10.1126/science.1202793.
28. Lebon, G.; Edwards, P.C.; Leslie, A.G.; Tate, C.G. Molecular Determinants of CGS21680 Binding to the Human Adenosine A2A Receptor. *Mol Pharmacol* **2015**, *87*, 907-915, doi:10.1124/mol.114.097360.
29. Dore, A.S.; Robertson, N.; Errey, J.C.; Ng, I.; Hollenstein, K.; Tehan, B.; Hurrell, E.; Bennett, K.; Congreve, M.; Magnani, F., et al. Structure of the adenosine A(2A) receptor in complex with ZM241385 and the xanthines XAC and caffeine. *Structure* **2011**, *19*, 1283-1293, doi:10.1016/j.str.2011.06.014.
30. Congreve, M.; Andrews, S.P.; Doré, A.S.; Hollenstein, K.; Hurrell, E.; Langmead, C.J.; Mason, J.S.; Ng, I.W.; Tehan, B.; Zhukov, A. Discovery of 1, 2, 4-triazine derivatives as adenosine A2A antagonists using structure based drug design. *J Med Chem* **2012**, *55*, 1898-1903, doi:10.1021/jm201376w.
31. Carpenter, B.; Nehme, R.; Warne, T.; Leslie, A.G.; Tate, C.G. Erratum: Structure of the adenosine A2A receptor bound to an engineered G protein. *Nature* **2016**, *538*, 542, doi:10.1038/nature19803.
32. Batyuk, A.; Galli, L.; Ishchenko, A.; Han, G.W.; Gati, C.; Popov, P.A.; Lee, M.Y.; Stauch, B.; White, T.A.; Barty, A., et al. Native phasing of x-ray free-electron laser data for a G protein-coupled receptor. *Sci Adv* **2016**, *2*, e1600292, doi:10.1126/sciadv.1600292.
33. Sun, B.; Bachhawat, P.; Chu, M.L.; Wood, M.; Ceska, T.; Sands, Z.A.; Mercier, J.; Lebon, F.; Kobilka, T.S.; Kobilka, B.K. Crystal structure of the adenosine A2A receptor bound to an antagonist reveals a potential allosteric pocket. *Proc Natl Acad Sci U S A* **2017**, *114*, 2066-2071, doi:10.1073/pnas.1621423114.
34. Martin-Garcia, J.M.; Conrad, C.E.; Nelson, G.; Stander, N.; Zatsepin, N.A.; Zook, J.; Zhu, L.; Geiger, J.; Chun, E.; Kissick, D., et al. Serial millisecond crystallography of membrane and soluble protein microcrystals using synchrotron radiation. *IUCrJ* **2017**, *4*, 439-454, doi:papers3://publication/doi/10.1107/S205225251700570X.
35. Melnikov, I.; Polovinkin, V.; Kovalev, K.; Gushchin, I.; Shevtsov, M.; Shevchenko, V.; Mishin, A.; Alekseev, A.; Rodriguez-Valera, F.; Borshchevskiy, V., et al. Fast iodide-SAD phasing for high-throughput membrane protein structure determination. *Science Advances* **2017**, *3*, e1602952, doi:papers3://publication/doi/10.1126/sciadv.1602952.
36. Cheng, R.K.Y.; Segala, E.; Robertson, N.; Deflorian, F.; Dore, A.S.; Errey, J.C.; Fiez-Vandal, C.; Marshall, F.H.; Cooke, R.M. Structures of Human A1 and A2A Adenosine Receptors with Xanthines Reveal Determinants of Selectivity. *Structure* **2017**, *25*, 1275-1285 e1274, doi:10.1016/j.str.2017.06.012.
37. Weinert, T.; Olieric, N.; Cheng, R.; Brunle, S.; James, D.; Ozerov, D.; Gashi, D.; Vera, L.; Marsh, M.; Jaeger, K., et al. Serial millisecond crystallography for routine room-temperature structure determination at synchrotrons. *Nat Commun* **2017**, *8*, 542, doi:10.1038/s41467-017-00630-4.
38. Broecker, J.; Morizumi, T.; Ou, W.L.; Klingel, V.; Kuo, A.; Kissick, D.J.; Ishchenko, A.; Lee, M.Y.; Xu, S.; Makarov, O., et al. High-throughput in situ X-ray screening of and data collection from protein crystals at room temperature and under cryogenic conditions. *Nat Protoc* **2018**, *13*, 260-292, doi:10.1038/nprot.2017.135.
39. Eddy, M.T.; Lee, M.Y.; Gao, Z.G.; White, K.L.; Didenko, T.; Horst, R.; Audet, M.; Stanczak, P.; McClary, K.M.; Han, G.W., et al. Allosteric Coupling of Drug Binding and Intracellular Signaling in the A2A Adenosine Receptor. *Cell* **2018**, *172*, 68-80 e12, doi:10.1016/j.cell.2017.12.004.
40. Rucktooa, P.; Cheng, R.K.Y.; Segala, E.; Geng, T.; Errey, J.C.; Brown, G.A.; Cooke, R.M.; Marshall, F.H.; Dore, A.S. Towards high throughput GPCR crystallography: In Meso soaking of Adenosine A(2A) Receptor crystals. *Sci Rep-Uk* **2018**, *8*, 41, doi:ARTN 4110.1038/s41598-017-18570-w.
41. Baker, F.N.; Porollo, A. CoeViz: a web-based tool for coevolution analysis of protein residues. *BMC bioinformatics* **2016**, *17*, 1.
42. Vilar, S.; Cozza, G.; Moro, S. Medicinal chemistry and the molecular operating environment (MOE): application of QSAR and molecular docking to drug discovery. *Curr Top Med Chem* **2008**, *8*, 1555-1572, doi:papers3://publication/uuid/F958CC2E-BFDB-4D0E-8D14-A40B6D7CA53D.

© 2018 by the authors. Submitted for possible open access publication under the terms and conditions of the Creative Commons Attribution (CC BY) license (http://creativecommons.org/licenses/by/4.0/).
